# Supplementary material for: Scaffold-Free Retinal Pigment Epithelium Microtissues Exhibit Increased Release of PEDF
Source: Int J Mol Sci. 2021 Oct 20;22(21):11317. doi: 10.3390/ijms222111317 (PMC8583603; doi:10.3390/ijms222111317)

Supplementary Figure S1: *Automated live-dead analysis of cell aggregates; code and example images.*

Image j custom macro designed and written by Dr. Mark Ungrin

```
macro "Analyse clusters [f5]" {
	inputImage=getTitle();
	print("\\Clear");
	run("Clear Results");
	roiManager("Reset");
	print("Processing "+inputImage);

	mask_threshold=1200;
	data_slice_1=3;
	data_slice_2=2;
	mask_slice=1;
	min_size=1000;
	smoothing_iterations=20; // used during erode-dilate cycles to smooth outlines of masks, controls how finely the watershed algorithm divides up clusters
	test_mask=false;
	run_watershed=true;
	blur_size=10; // how much to blur the blue image before generating the mask - makes watershed less aggressive about splitting things up

	Dialog.create("Preferences");
	sliceCount = nSlices;
	sliceChoices=newArray(sliceCount);

//	Dialog.addNumber("Slice to use to identify objects:", mask_slice);
	for (i=0; i<sliceCount; i++) {
		Dialog.addString("Name for slice "+(i+1)+":", "Slice_"+(i+1));
		sliceChoices[i]=""+(i+1);
	}
	Dialog.addChoice("Slice to use to identify objects:", sliceChoices);
	//Dialog.addNumber("Slice to use to identify objects:", mask_slice);
	Dialog.addCheckbox("Only test object identification?", false);
	Dialog.addCheckbox("Run watershed segmentation to break apart objects in contact?", true);
	Dialog.addCheckbox("Automatically identify threshold value?", true);
	Dialog.addNumber("Manual threshold (used only if NOT selected automatically):", 1200);
	Dialog.addNumber("Smoothing iterations on mask:", smoothing_iterations);
	Dialog.addNumber("Blur levels on mask:", blur_size);
	Dialog.addNumber("Minimum object size to analyse:", min_size);
	Dialog.show();

	sliceNames=newArray(sliceCount);
	for (i=0; i<sliceCount; i++) {
		sliceNames[i]=Dialog.getString();
		print("Name for slice "+(i+1)+": "+sliceNames[i]);
	}
	mask_slice = Dialog.getChoice();
	print("mask_slice "+mask_slice);
	test_mask = Dialog.getCheckbox();
	print("test_mask "+test_mask);
	run_watershed = Dialog.getCheckbox();
```

```
	print("run_watershed "+run_watershed);
	auto_threshold = Dialog.getCheckbox();
	print("auto_threshold "+auto_threshold);
	mask_threshold = Dialog.getNumber();
	print("mask_threshold "+mask_threshold);
	smoothing_iterations = Dialog.getNumber();
	print("smoothing_iterations "+smoothing_iterations);
	blur_size = Dialog.getNumber();
	print("blur_size "+blur_size);
	min_size = Dialog.getNumber();
	print("min_size "+min_size);

	function check_saturation(name) {
		bit_depth=bitDepth();
		getRawStatistics(nPixels, meanVal, minVal, maxVal, stdVal, histogram);
		if (maxVal == (pow(2,bit_depth)-1)) {
			saturated_pixels = histogram[maxVal]/nPixels;
		   getLut(reds, greens, blues);
		   reds[255] = 255-reds[255];
		   greens[255] = 255-greens[255];
		   blues[255] = 255-blues[255];
		   setLut(reds, greens, blues);
			showMessage("WARNING("+name+"): Intensity values for "+histogram[maxVal]+" pixels out of "+nPixels+" are saturated (value="+maxVal+")\nIntegrated and mean intensity values calculated in this channel will be artefactually low.\nConduct your analyses accordingly\nPlease try to capture images without significant numbers of saturated pixels.");
		}
	}

	for (i=0; i<sliceCount; i++) {
		selectWindow(inputImage);
		setSlice(i+1);
		check_saturation(sliceNames[i]);
		run("Duplicate...", "title="+sliceNames[i]);
	}
	selectWindow(inputImage);
	setSlice(mask_slice);
	run("Duplicate...", "title=MASK_SLICE");

	if (blur_size>0) run("Gaussian Blur...", "sigma="+blur_size+" scaled");

	if (auto_threshold) {
		setAutoThreshold("Default dark");
	} else {
		setThreshold(mask_threshold, 65535);
	}

	setOption("BlackBackground", false);
	run("Convert to Mask");
	run("Options...", "iterations="+smoothing_iterations+" count=5 pad do=Nothing");
	run("Fill Holes");
	setOption("BlackBackground", false);
	run("Dilate");
```

```
	run("Erode");
	//run("Dilate");
	if (run_watershed) run("Watershed");
	//run("Set Measurements...", "area mean standard min centroid fit shape integrated display redirect=None 
decimal=3");
	run("Set Measurements...", "area mean standard min centroid shape integrated display redirect=None 
decimal=3");
	run("Input/Output...", "jpeg=85 gif=-1 file=.csv use use_file copy_column save_column");

	run("Clear Results");
	roiManager("Reset");

	run("Analyze Particles...", "size="+min_size+"-Infinity show=Nothing display exclude add");
	selectWindow("MASK_SLICE");
	run("Close");
	run("Clear Results");
	if (!test_mask) {
		setBatchMode(true);
		roi_count=roiManager("Count");
		meanArray=newArray(sliceCount);
		print("sliceCount "+sliceCount+", roi_count "+roi_count);
		for (i=0; i<roi_count; i++){
			roiManager("Select", i);
			roiManager("Rename", "Aggregate #"+(i+1)+"("+inputImage+")");
			for (j=0; j<sliceCount; j++){
				selectWindow(sliceNames[j]);
				print("ROI "+i+", slice "+sliceNames[j]+" of "+sliceCount);
				roiManager("Measure");
				meanArray[j]=getResult("Mean");
			}

			summaryRow = nResults();
			setResult("Label", summaryRow, "Aggregate #"+(i+1)+"("+inputImage+")");
			for (j=0; j<sliceCount; j++){
				setResult(sliceNames[j], summaryRow, meanArray[j]);
			}
			setResult("SUMMARY", summaryRow, "SUMMARY");
			updateResults();
		}
	}
	selectWindow(inputImage);
	imgW = getWidth();
	imgH = getHeight();

	setBatchMode(false);

	montageName = "Montage ("+inputImage+")";
	newImage(montageName, "RGB black", imgW*sliceCount, imgH, 1);
	setBatchMode(true);
	fontSize = imgH / 10;
	setFont("Sanserif", fontSize);
	setColor(255, 255, 255);
	for (j=0; j<sliceCount; j++){
```

```
            selectWindow(sliceNames[j]);
            run("Select All");
            run("Copy");
            selectWindow(montageName);
            makeRectangle(imgW*j, 0, imgW, imgH);
            run("Paste");
            drawString(sliceNames[j], imgW*j+fontSize*0.5, fontSize*1.5);
            selectWindow(sliceNames[j]);
            run("Close");
        }
        setBatchMode(false);

        selectWindow(inputImage);
        roiManager("Show All");

        selectWindow("Results");

        selectWindow(montageName);
        roiManager("Show All");
}
```

<u>Example 1: R400 RPE-μT control</u>

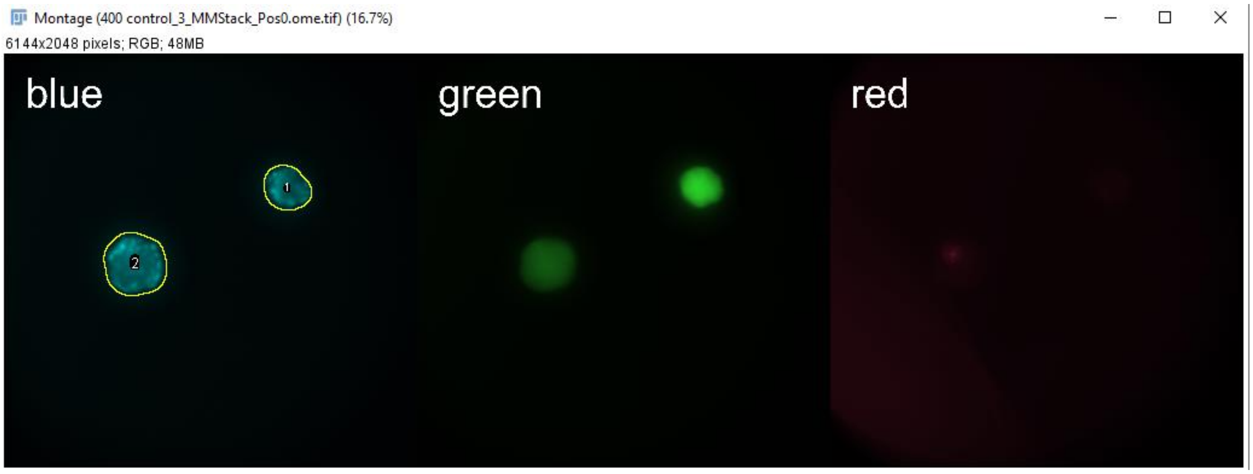

<u>Example 2: R100 RPE-μT injected:</u>

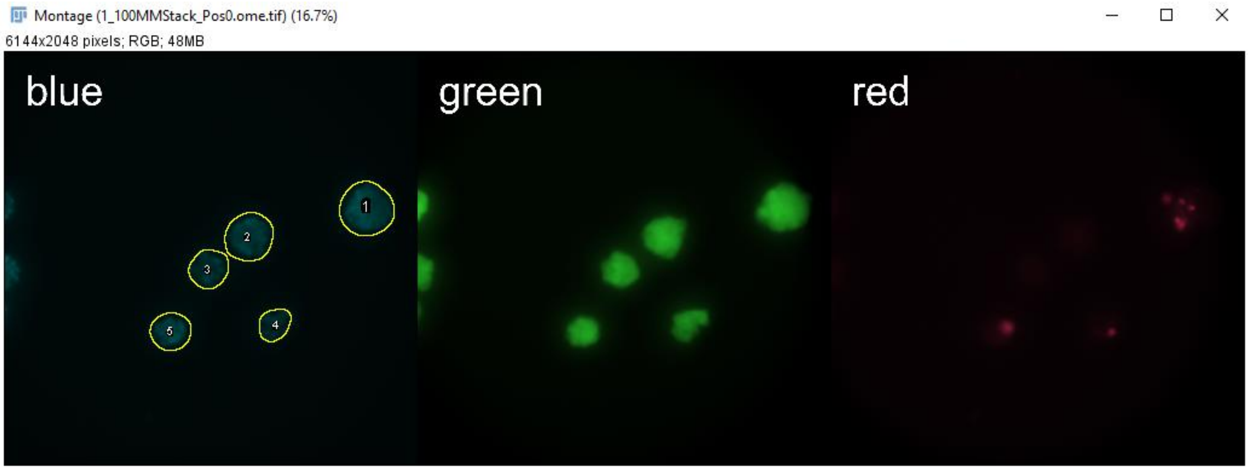

Supplement: Supplementary file 1 [file ijms-22-11317-s001.zip › ijms-1384645-Figure S1.pdf]
