# Supplementary material for: Scaffold-Free Retinal Pigment Epithelium Microtissues Exhibit Increased Release of PEDF
Source: Int J Mol Sci. 2021 Oct 20;22(21):11317. doi: 10.3390/ijms222111317 (PMC8583603; doi:10.3390/ijms222111317)
Supplement: Supplementary file 1 [file ijms-22-11317-s001.zip › ijms-1384645-Table S1.pdf]

Table S1 *A list of antibodies used in this project, the animals they were raised in, their optimized concentrations, and their specificity and function.*

Antibody	Animal	Manufacturer	Conc.	Specificity and function	Reference
**CRALBP**	Mouse	Abcam cat # ab15051	1/500	An essential protein for the visual recycling function of RPE	[49, 50]
**melanopsin**	Rabbit	Advanced Targeting Systems cat # Ab-n38	1/500	Allows RPE to absorb light to protect the retina against photo-oxidation	[11]
**RPE65**	Mouse	Abcam cat # ab13826	1/500	An essential isomerohydrolase enzyme in the retinoid visual cycle	[102]
**Sox9**	Rabbit	Millipore cat # ab5535	1/500	Transcription factor that regulates the expression of important genes in RPE like BEST1 and RPE65	[103, 104]
**ZO-1**	Mouse	Invitrogen cat # 339100	1/50	Tight junction marker is reported to regulate RPE homeostasis	[105]
**LRAT**	Rabbit	Abcam cat # ab137304	1/500	An essential protein for the retinol uptake by RPE and 11-cis retinal recycling	[51, 102]
**β-catenin**	Rabbit	Cell Signalling cat # 8814	1/500	Signalling molecule that is linked to inducing the expression of Mitf and OTX2 in development	[106]
**Best1**	Mouse	Abcam cat # ab2182	1/500	Found in the basolateral side of the RPE and preserves calcium ion regulation	[52, 53]
**MITF**	Rabbit	Abcam cat # ab20663	1/500	RPE specific transcription factor that is crucial for RPE differentiation and function	[55]
**Laminin**	Rabbit	Abcam cat # ab11575	1/200	Extracellular matrix secreted by RPE apically and basally contribute to the structure of the basement membrane and interphotoreceptor matrix.	[56, 57]
**Fibronectin**	Rabbit	Abcam cat # ab23750	1/200	As per laminin	

**Collagen IV**	Rabbit	Abcam<br>cat # ab6586	1/200	As per laminin	

Additional supplemental references related to Table S1.

102. Moiseyev, G.; Chen, Y.; Takahashi, Y.; Wu, B.X.; Ma, J.; Nathans, J. RPE65 visual is the cycle in the isomerohydrolase retinoid. *Proc. Natl. Acad. Sci. U. S. A.* **2005**, *102*, 12413–12418, doi:10.1073/pnas.0503460102.

103. Masuda, T.; Esumi, N. SOX9, through Interaction with Microphthalmia-associated Transcription Factor (MITF) and OTX2, Regulates BEST1 Expression in the Retinal Pigment Epithelium. *J. Biol. Chem.* **2010**, *285*, 26933–26944, doi:10.1074/jbc.M110.130294.

104. Masuda, T.; Wahlin, K.; Wan, J.; Hu, J.; Maruotti, J.; Yang, X.; Iacovelli, J.; Wolkow, N.; Kist, R.; Dunaief, J.L.; et al. Transcription Factor SOX9 Plays a Key Role in the Regulation of Visual Cycle Gene Expression in the Retinal Pigment Epithelium. *J. Biol. Chem.* **2014**, *289*, 12908–12921, doi:10.1074/jbc.M114.556738.

105. Georgiadis, A.; Tschernutter, M.; Bainbridge, J.W.B.; Balaggan, K.S.; Mowat, F.; West, E.L.; Munro, P.M.G.; Thrasher, A.J.; Matter, K.; Balda, M.S.; et al. The tight junction associated signalling proteins ZO-1 and ZONAB regulate retinal pigment epithelium homeostasis in mice. *PLoS One* **2010**, *5*, 1–10, doi:10.1371/journal.pone.0015730.

106. Fujimura, N.; Taketo, M.M.; Mori, M.; Korinek, V.; Kozmik, Z. Spatial and temporal regulation of Wnt/β-catenin signaling is essential for development of the retinal pigment epithelium. *Dev. Biol.* **2009**, *334*, 31–45, doi:10.1016/j.ydbio.2009.07.002.
