# Supplementary material for: Scaffold-Free Retinal Pigment Epithelium Microtissues Exhibit Increased Release of PEDF
Source: Int J Mol Sci. 2021 Oct 20;22(21):11317. doi: 10.3390/ijms222111317 (PMC8583603; doi:10.3390/ijms222111317)
Supplement: Supplementary file 1 [file ijms-22-11317-s001.zip › ijms-1384645-Tables S2-S3.pdf]

Table S2 *RT-qPCR data comparing gene expression in ARPE-19 RPE-µT to adherent culture.*

Gene	Adherent culture mean ΔCt ± SD	RPE-µT mean ΔCt ± SD	P-value
FASL	7.01 ±1.24	6.26 ±1.50	0.0161
TIMP3	-3.23 ±2.94	-2.93 ±3.73	0.569
TGFB	-2.86 ±1.57	-0.806 ±1.76	0.0024
VEGF-A	-3.12 ±1.67	0.239 ±1.61	0.0049
PDGF-AA	-0.476 ±0.841	0.392 ±0.815	0.0005
IGF-1	5.04 ±3.31	3.25 ±3.66	0.0322
BDNF	-4.28 ±1.18	-3.55 ±1.589	0.206
GAS6	-0.758 ±0.849	0.854 ±0.530	0.002
FGF2	-5.22 ±1.02	-2.66 ±2.49	0.0002
RPE65	1.54 ±0.878	0.749 ±0.447	0.109
CFH	-1.69±0.804	-1.97 ±1.55	0.455
TRMP1	1.01 ±0.351	0.659 ±0.769	0.193
BEST1	0.367 ±0.752	0.190 ±0.459	0.519
FGF2R	0.852 ±0.308	0.895 ±0.244	0.931
MYRIP	-0.397 ±0.414	-0.071 ±0.482	0.151
CCL2	-5.46 ±1.73	-4.01 ±2.31	0.008
IL-8	-4.91 ±1.64	-1.83 ±1.66	0.0005
LHX2	0.0847 ±1.73	3.77 ±2.23	0.0005
LOXL	-4.47 ±0.856	-1.72 ±1.26	0.0002
KDR	3.68 ±2.52	7.18 ±2.30	0.001

Table S3 *RT-qPCR data comparing gene expression in ES-RPE RPE-µT to adherent culture.*

**Gene**	Mean ΔCt ± SD ES-RPE adherent culture	Mean ΔCt ± SD ES-RPE uT	P-value
FASL	7.31 ±0.497	7.38 ±0.638	0.700
TIMP3	-5.50 ±1.11	-3.82 ±3.91	0.037
TGFB	-0.886 ±1.04	0.473 ±0.714	0.001
VEGF-A	-2.87 ±1.28	2.54 ±3.02	0.002
PDGF-AA	0.730 ±0.476	0.560 ±0.303	0.232
IGF-1	2.96 ±1.08	4.91 ±2.51	0.0068
BDNF	0.453 ±4.06	2.01 ±4.27	0.0049
GAS6	-0.573 ±0.476	0.167 ±0.488	0.032
FGF2	-1.22 ±1.22	0.877 ±3.06	0.0098
RPE65	0.66 ±0.646	-0.152 ±0.986	0.024
CFH	-3.04 ±1.314	-0.599 ±1.12	0.001
TRMP1	-0.433 ±2.57	-0.439 ±1.14	0.638
BEST1	-0.157 ±0.977	-0.229 ±1.53	>0.99
FGF2R	-0.217 ±0.270	-0.269 ±0.359	0.625
MYRIP	-1.14 ±0.796	-0.776 ±0.830	0.042
CCL2	-3.99 ±3.50	-0.25 ±4.41	0.240
IL-8	-1.59 ±2.47	3.77 ±3.58	0.001
LHX2	-0.834 ±1.79	6.18 ±2.36	0.001
LOXL	-3.67 ±1.05	-0.72 ±0.590	0.001
KDR	0.461 ±2.03	3.08 ±2.41	0.002
